# Monitoring of cerebral autoregulation during major surgery in adults, including cardiac surgery: mechanisms, anesthetic impacts, and clinical applications

**DOI:** 10.3389/fmed.2026.1782247

**Published:** 2026-08-13

**Authors:** Boxiong Gao, Abdulrahman Khaled Alwesabi, Bokang Yang, Jinxiang Xie, Fang Li, Xiaodong Su, Qijing Liu, Ying Liu, Qian Fu, Chengying Ji, Zhaohui Gao, Jiayi Xie, Yatao Liu

**Affiliations:** 1 The First School of Clinical Medicine, Lanzhou University, Lanzhou, Gansu, China; 2 Department of Anesthesiology and Surgery, First Hospital of Lanzhou University, Lanzhou, Gansu, China

**Keywords:** anesthetics, blood pressure management, cerebral autoregulation, monitoring, perioperative

## Abstract

**Background:**

Cerebral autoregulation (CA) is a critical neuroprotective mechanism that maintains stable cerebral blood flow across varying blood pressure (BP). The perioperative period disrupts CA through anesthetic and surgical interventions, yet standardized monitoring protocols are lacking in adult major surgery.

**Findings:**

Evidence demonstrates that anesthetic choice significantly modulates CA. Total intravenous anesthesia better preserves autoregulation compared to volatile agents. Surgical factors—including cardiopulmonary bypass, patient positioning, and procedure duration—further influence CA integrity. Near-infrared spectroscopy-derived cerebral oximetry index (COx) provides the most practical approach for routine perioperative CA monitoring. Personalized BP management based on autoregulation thresholds optimizes cerebral perfusion and reduces neurological complications, as shown in cardiac surgery.

**Conclusion:**

Integrating CA-preserving anesthetic techniques with real-time monitoring represents a promising strategy for precision perioperative care. Future validation through large-scale randomized trials is essential to establish standardized protocols and confirm effects on patient-centered outcomes.

## 1 Introduction

The maintenance of cerebral function is critically dependent on a tightly regulated supply of cerebral blood flow (CBF), which delivers oxygen and nutrients while removing metabolic wastes. This regulation is masterfully achieved by the mechanism of cerebral autoregulation (CA), which maintains a relatively constant CBF across a range of systemic BP. CA is conventionally assessed in two complementary dimensions: static CA, which reflects the steady-state pressure-flow relationship, and dynamic CA, which describes the rapid cerebrovascular response to transient BP changes (1). The perioperative period poses distinct challenges to both facets of CA through multiple mechanisms, including anesthetic-mediated vasodilation, surgical stress responses, hemodynamic fluctuations, and metabolic alterations. These perturbations can significantly compromise neurological outcomes, particularly in vulnerable patient populations. Impaired CA is consequently associated with a spectrum of adverse postoperative outcomes, including stroke, delirium, cognitive dysfunction, and other end-organ injuries (2–4). Despite its established clinical significance, standardized protocols for monitoring CA in the perioperative setting remain elusive. This review focuses only on adult patients undergoing major surgeries, including cardiac, vascular, neurosurgical, and large thoraco-abdominal surgeries. Neonates, children, and patients with severe brain injuries are not within the scope of discussion. There are two reasons: (1) Cerebral autoregulation (CA) in the immature or damaged brain is fundamentally different from that in mature, uninjured adults; (2) The peri-operative management principles for these special populations deserve a dedicated review. Our overarching goal is to translate contemporary evidence into practical strategies for perioperative management.

## 2 Clinical significance of cerebral autoregulation in the perioperative period

CA serves a vital protective function in the cerebral circulation, preventing both hypoperfusion during hypotension and cerebral overperfusion during hypertension. Although the clinical application of CA monitoring has been predominantly established in neurocritical care, its utility is increasingly recognized in surgical settings (2). Historically, high-level evidence linking CA monitoring to postoperative outcomes was largely confined to cardiac surgery. However, recent investigations have demonstrated its expanding relevance to non-cardiac surgeries. For instance, a recent meta-analysis indicated that optimizing intraoperative management based on near-infrared spectroscopy (NIRS)-derived regional cerebral oxygen saturation (rcSO₂) significantly reduces the risk of early postoperative cognitive dysfunction after elective non-cardiac procedures (5). Furthermore, a retrospective study that directly compared NIRS-based and transcranial Doppler (TCD)-based CA monitoring during elective neurosurgery confirmed the feasibility of deriving individualized blood pressure (BP) targets from both modalities in this specific surgical population (6). In the surgical context, prolonged exposure of the brain to a mean arterial pressure (MAP) below the lower limit of autoregulation (LLA) is correlated with an elevated risk of complications, including prolonged mechanical ventilation, acute kidney injury, and mortality (7).

## 3 Monitoring strategies for perioperative cerebral autoregulation

An optimal monitoring strategy for CA should be non-invasive, technically straightforward, and accurate. Most validated methodologies are based on time-domain or frequency-domain analyses that quantify the relationship between cerebral perfusion pressure (or its surrogate, MAP) and surrogates of CBF or cerebral blood volume.

### 3.1 Transcranial Doppler-based methods: transfer function analysis, ARI and mx

Transcranial Doppler (TCD) measures middle cerebral artery flow velocity (MCAv) as a surrogate for CBF. This interpretation is predicated on the critical assumption that the diameter of the insonated vessel remains constant throughout the measurement period—an assumption that may be challenged by changes in CO₂ tension, hypoxia, or the administration of certain anesthetic agents (1). Transfer function analysis is a frequency-domain method that estimates dynamic CA from spontaneous oscillations in MAP (input) and MCAv (output). Three key parameters are derived: coherence (strength of linear relationship), gain (amplitude of flow response), and phase (temporal lead/lag of flow relative to pressure). In the low-frequency range (0.07–0.20 Hz), increased phase and decreased gain indicate improved dynamic CA (8). Autoregulation Index (ARI) is a time-domain model that compares the MCAv response to a rapid MAP change (e.g., thigh cuff deflation) against 10 theoretical step-response curves (0 = absent, 9 = most intact) (9). Mean Flow Index (Mx) is calculated as the moving correlation coefficient between slow waves of MCAv and CPP (or MAP). An Mx > 0.3 typically indicates impaired CA (10).

### 3.2 Near-infrared spectroscopy-based method: COx

The Cerebral Oximetry Index (COx) provides a non-invasive assessment of dynamic CA by calculating the Pearson correlation coefficient between spontaneous slow waves of MAP and regional cerebral oxygen saturation (rScO₂) measured by near-infrared spectroscopy (NIRS). When CA is intact, COx ≈ 0; when impaired, COx → +1. A threshold COx > 0.3 is commonly used to define impaired CA. The MAP values at which COx crosses this threshold define the lower and upper limits of autoregulation (LLA and ULA), and the MAP with the minimum COx is the optimal MAP (OptMAP) (11). In practice, LLA and ULA are derived by plotting COx against MAP bins (e.g., every 5 mmHg) and fitting a smoothing curve (e.g., LOESS or polynomial regression). The LLA is the MAP below which COx consistently exceeds 0.3, indicating pressure-passive cerebral perfusion; the ULA is the corresponding upper threshold. The MAP at which COx reaches its minimum represents OptMAP, the point of most efficient autoregulation. A conceptually similar index, the Hemoglobin Volume Index (HVx), derived from the correlation between MAP and relative total hemoglobin, can also be applied for CA assessment (2, 12).

### 3.3 Intracranial pressure-based method: PRx

The Pressure Reactivity Index (PRx) is a continuous metric of cerebrovascular reactivity, calculated as the moving Pearson correlation between intracranial pressure (ICP) and arterial blood pressure (ABP) over a standard 5-min window. A PRx < 0 (typically negative) indicates intact CA, whereas PRx → +1 indicates impaired CA (ICP passively follows ABP). Although PRx requires invasive ICP monitoring, it has been validated in elective neurosurgery, for example in patients undergoing craniotomy for tumor resection or those with space-occupying lesions, where it can guide individualized cerebral perfusion pressure (CPP) management (13).

### 3.4 Clinical implementation

The COx method offers a non-invasive, practical approach for routine perioperative CA monitoring. TCD-based indices provide high temporal resolution but are operator-dependent. PRx remains invaluable in neurocritical care and selected neurosurgical cases where ICP is already monitored.

## 4 Impact of anesthetic methods on cerebral autoregulation

**Figure 1:**
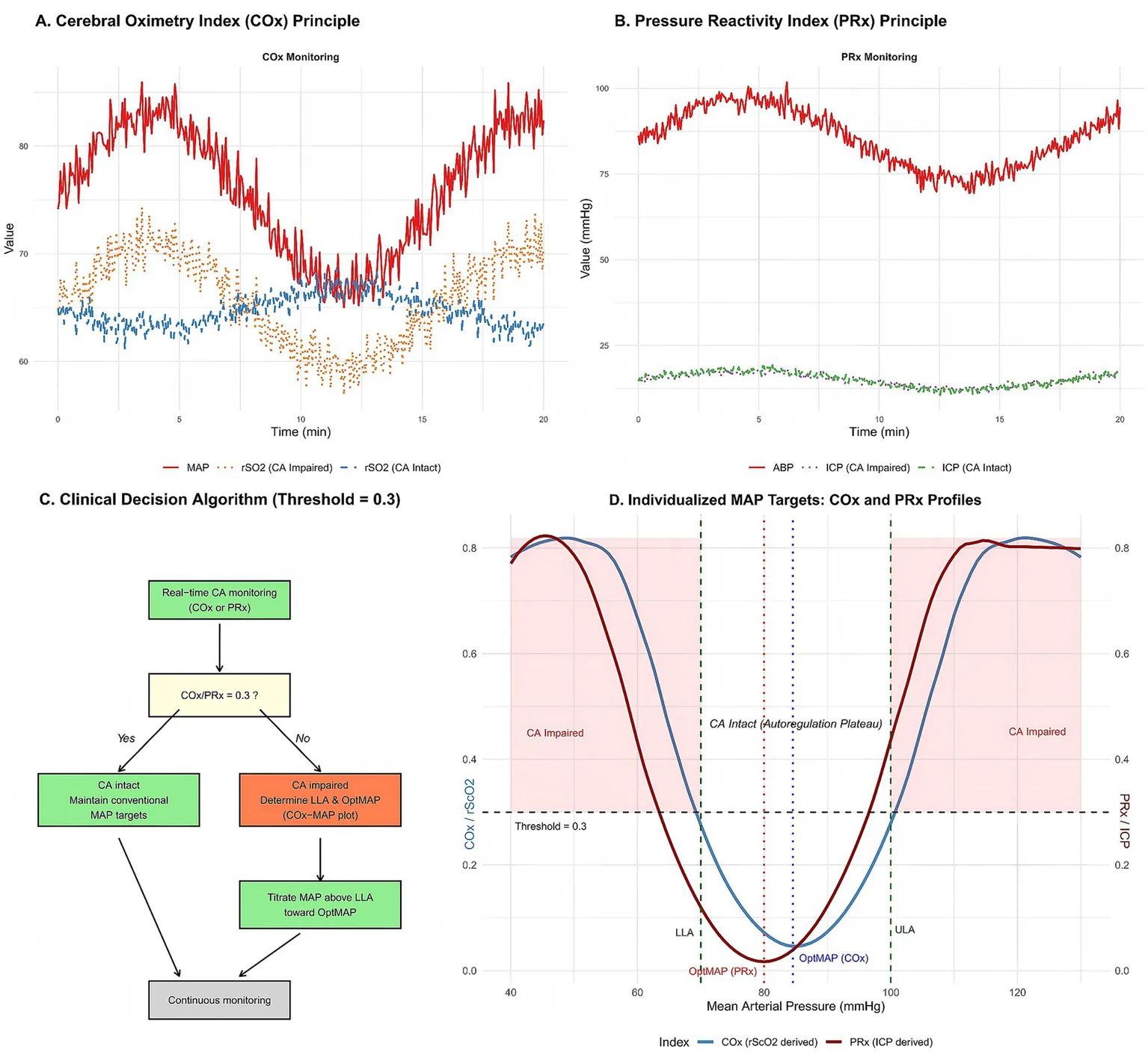
Principles and clinical application of cerebral oximetry index (COx) and pressure reactivity index (PRx) for perioperative cerebral autoregulation monitoring. **(A)** COx principle. COx is calculated as the Pearson correlation between slow-wave oscillations in MAP and rScO₂. When CA is intact, COx ≈ 0; when impaired, COx > 0.3 (positive correlation). **(B)** PRx principle. PRx is calculated as the Pearson correlation between slow-wave oscillations in ABP and ICP. Intact CA yields PRx < 0.3; impaired CA yields PRx > 0.3. **(C)** Clinical decision algorithm. Real-time COx or PRx guides management: If ≤ 0.3, CA is intact → maintain conventional MAP targets. If > 0.3, CA is impaired → determine LLA and OptMAP, then titrate MAP above LLA toward OptMAP. **(D)** Individualized MAP targets. Plot COx (or PRx) against MAP bins. The minimum of the fitted curve defines OptMAP; the points where the curve crosses 0.3 define LLA and ULA. Shaded red areas indicate MAP ranges with impaired CA. COx, cerebral oximetry index; PRx, pressure reactivity index; MAP, mean arterial pressure; rScO₂, regional cerebral oxygen saturation; ABP, arterial blood pressure; ICP, intracranial pressure; LLA, lower limit of autoregulation; OptMAP, optimal mean arterial pressure.

The blood–brain barrier (BBB) and hepatic first-pass metabolism significantly restrict the central effects of many systemically administered drugs on the cerebral vasculature. The route of drug administration is a critical determinant of drug distribution and plasma concentration, thereby variably influencing (CBF) and its regulation. Evidence from non-human primate models indicates distinct differential effects among anesthetic agents. Under combined fentanyl and isoflurane anesthesia, only fentanyl was associated with a classic static CA curve. Assessments of dynamic autoregulation using the pressure reactivity index (PRx) revealed that both static and dynamic CA were impaired during high-dose isoflurane anesthesia (14). In contrast, a regimen of low-dose isoflurane combined with fentanyl successfully preserved autoregulatory function. This dichotomy between intravenous and volatile anesthetics is corroborated by clinical studies in humans. Total intravenous anesthesia (TIVA) with a propofol-sufentanil combination, while reducing systemic BP, does not impair static CA. During the induction phase, patients receiving sevoflurane inhalation anesthesia exhibit a decline in CA indices compared to those under propofol-remifentanil TIVA, with the TIVA technique demonstrating superior preservation of autoregulation. Furthermore, propofol-fentanyl TIVA maintains dynamic CA, whereas sevoflurane inhalation anesthesia impairs it—a finding consistently observed across adult and pediatric populations (15–17). In these studies, Juhász et al. (15) enrolled 27 patients undergoing general anesthesia with propofol-sufentanil TIVA, and reported preserved cerebral autoregulation and CO₂ reactivity; Conti et al. (16) randomly allocated 40 patients with spinal or maxillo-facial disorders to propofol-remifentanil versus sevoflurane, and found that high-dose sevoflurane impaired autoregulation whereas propofol-remifentanil preserved it; Jildenstål et al. (17) studied 15 infants undergoing craniofacial surgery under sevoflurane, and observed pressure-dependent cerebral perfusion indicating impaired autoregulation. Consequently, inhalation anesthesia appears more likely to disrupt CA compared to TIVA. Beyond primary anesthetic techniques, interventions like direct stellate ganglion blockade have been shown to significantly increase ipsilateral CBF, although their precise impact on CA metrics remains an area for further investigation (18). To date, no other anesthetic techniques have been conclusively identified to directly influence CA. A paramount consideration in clinical practice is that the effects of any anesthetic method on CA are not uniform. These effects must be interpreted in the context of patient-specific factors, including pre-existing cerebral comorbidities and concurrently administered perioperative medications, all of which can significantly modulate individual autoregulatory responses (Figure 1).

## 5 Impact of anesthetic agents on cerebral autoregulation

Pharmacological interventions in the perioperative period modulate CBF mainly via two drug classes: anesthetic and vasoactive agents. In clinical practice, anesthesia is rarely achieved with a single agent, necessitating that studies on drug-induced CA alterations carefully consider complex drug interactions and potential confounding variables (See Table 1 for a systematic summary).

**Table 1:** Effects of anesthetic and vasoactive agents on cerebral autoregulation.

Category	Specific agent	Effect on CA	Mechanism	Evidence level	Clinical recommendation
Intravenous anesthetics	Propofol	Preserves	GABA-A receptor agonism; maintains pressure-flow coupling	Strong (Human)	Preferred for CA maintenance; suitable for neuroanesthesia
	Benzodiazepines (Midazolam)	Preserves/May enhance dynamic CA	GABA-A receptor modulation; reduces CBF pressure passivity	Moderate (Human)	Suitable for CA preservation; may benefit dynamic CA
	Ketamine	Preserves	NMDA receptor antagonism; maintains autoregulatory curve	Moderate (Human/Animal)	Safe regarding CA function; useful in combination regimens
	Etomidate	Unknown (Limited data)	GABA-A receptor activation; reduces CBF and ICP	Limited	Insufficient evidence; use when hemodynamic stability paramount
Inhaled anesthetics	Sevoflurane	Impairs (Dose-dependent)	Dose-related vasodilation; disrupts neurovascular coupling (13)	Conflicting (Human/Animal)	Use with caution at >1 MAC; consider lower concentrations
	Isoflurane	Impairs (Dose-dependent)	Cerebral vasodilation; metabolic uncoupling (65)	Strong (Human/Animal)	Higher doses compromise CA; monitor in vulnerable patients
	Desflurane	Impairs	Similar to isoflurane; rapid onset/offset	Moderate (Human)	Avoid in patients with known CA impairment
	Nitrous Oxide (N₂O)	Preserves (when used alone)	NMDA receptor antagonism; minimal effect on CA plateau	Moderate (Primate)	Safe when used alone; combination effects require caution
Adjuvants	Dexmedetomidine	Preserves static CA/May impair dynamic CA	α2-adrenergic agonism; differential effects on static vs. dynamic CA (66)	Conflicting (Human studies)	Requires careful titration; monitor dynamic CA if used
Opioids	Remifentanil	Minimal effect	μ-opioid receptor agonism; minimal direct cerebrovascular effect	Moderate (Human)	Favorable CA profile; ideal for TIVA combinations
	Fentanyl/Sufentanil	Minimal effect	μ-opioid receptor activation; hemodynamic effects indirect	Moderate (Human)	Minimal direct CA impact; suitable across anesthetic techniques
Vasoactive agents	Norepinephrine	Increases CBF if CA impaired	*α*-adrenergic vasoconstriction; may improve perfusion pressure	Moderate (Meta-analysis)	First-line vasopressor when CA concern; improves perfusion
	Dopamine	Impairs (Dose-dependent in pediatrics)	Dopaminergic and adrenergic effects; dose-dependent impairment	Moderate (Human peds/Animal)	Caution in pediatric patients; prefer alternatives in adults
	Phenylephrine	Minimal direct effect	Pure α1-adrenergic agonism; primarily peripheral vasoconstriction	Moderate (Human)	Suitable for MAP elevation without direct CA effects

This table summarizes the available evidence on the differential effects of common perioperative agents on cerebral autoregulation (CA). The “Effect on CA” represents a consensus based on current literature. “Evidence Strength & Consistency” integrates the quality and agreement across studies: Strong, Consistent indicates support from multiple high-quality human studies; Moderate, Consistent indicates support from several studies but with some limitations; Conflicting indicates disparate findings in the literature; Limited Evidence indicates a conclusion based on few or preliminary studies. Clinical recommendations are derived from the synthesized evidence. CA, cerebral autoregulation; CBF, cerebral blood flow; ICP, intracranial pressure; MAP, mean arterial pressure; TIVA, total intravenous anesthesia; LLA, lower limit of autoregulation; MAC, minimum alveolar concentration.

### 5.1 Propofol

Propofol is a widely employed intravenous anesthetic known for its dose-dependent sedative properties. Evidence consistently demonstrates its capacity to preserve CA across diverse clinical scenarios. For instance, during sufentanil-based anesthesia, propofol maintains intact CA despite significant reductions in systemic MAP, with no consequential changes in central aortic or peripheral pulse pressures. This preservation of autoregulatory function extends to high-dose propofol administration and procedures such as robot-assisted prostatectomy (15, 19) The robustness of this effect is further highlighted in vulnerable populations; in preterm neonates undergoing propofol-assisted intubation, although the drug can induce hypotension and reduce cerebral activity, intact CA was maintained in the vast majority (95.5%) of patients (20). Collectively, these findings underscore propofol’s reliable ability to preserve CA under clinically relevant conditions, even in the presence of transient hemodynamic alterations.

### 5.2 Benzodiazepines

Benzodiazepines (BZDs) produce anxiolytic, amnesic, and sedative effects. Their impact on cerebrovascular regulation appears distinct from their sedative properties. This is illustrated by the administration of flumazenil, a BZD antagonist, which effectively reverses midazolam-induced sedation but fails to restore baseline CBF velocities or correct midazolam-induced alterations in cerebral circulation (21). A comparative randomized study in healthy subjects revealed that while both midazolam and propofol reduced steady-state CBF velocity, only midazolam significantly decreased transfer function gain in the low-frequency range (22). This specific finding indicates a diminished transmission of systemic MAP oscillations to CBF fluctuations, suggesting that midazolam may potentially enhance dynamic CA by reducing pressure-driven CBF variability.

### 5.3 Ketamine

Ketamine is a dissociative anesthetic with potent analgesic properties. Contrary to early reports suggesting it elevates intracranial pressure (ICP), contemporary evidence consistently demonstrates no significant impact of ketamine on ICP (23). Recent randomized controlled trials indicate that ketamine increases CBF in a region-specific manner (24). Critically, ketamine preserves CA across a range of doses in animal models, although low-dose administration may shift the autoregulatory curve toward higher MAP values. This CA-preserving property is similarly observed in human studies, confirming that ketamine does not impair autoregulatory function (25, 26).

### 5.4 Etomidate

Etomidate is an intravenous anesthetic characterized by its reduction of both ICP and CBF, without significantly altering cerebral perfusion pressure (CPP) (27). A notable gap in the current literature is the absence of studies specifically designed to investigate its effects on CA. Consequently, further research is essential to clarify the impact of etomidate on CA and neurological outcomes, particularly in vulnerable populations such as the elderly or patients with pre-existing cerebrovascular disease.

### 5.5 Volatile anesthetics

Volatile anesthetics are administered via inhalation and exert their central nervous system effects following pulmonary absorption and systemic distribution. A hallmark of modern halogenated agents is the dose-dependent suppression of cerebral metabolic rate, which is, however, frequently accompanied by an increase in CBF (28). The net effect of sevoflurane on CBF remains a subject of debate, and its impact on CA presents a similarly conflicting picture (29). Animal studies typically demonstrate preserved CA at 1.0 MAC but consistent impairment at 2.0 MAC (30). Human studies have yielded disparate results: some report no significant effect of sevoflurane on CA, even in cohorts with pre-existing impairment, while others suggest a narrowing of the autoregulatory plateau at clinically relevant concentrations (1.0 MAC) (31, 32). Sophisticated assessments of dynamic CA have revealed impairment specifically within the very low-frequency range, despite preserved function at higher frequencies (33).

This variability extends to other volatile agents. Isoflurane reliably impairs CA in humans in a dose-dependent manner, a effect shared by desflurane and halothane (14, 34, 35). Nitrous oxide (N₂O), an NMDA receptor antagonist, increases CBF regionally yet appears to preserve CA when used alone, as evidenced by primate studies (36, 37). However, its effects when combined with other volatiles are less clear and warrant further investigation. Xenon, another NMDA antagonist, is notable for its rapid onset and offset. Animal studies suggest that it maintains CA even at high concentrations, but a definitive confirmation from human trials is presently lacking (38). Mainly because of its high cost in clinical use and difficulty in production, which are limitations. It is hoped that its impact on CA can be clarified in the future.

### 5.6 Dexmedetomidine

Dexmedetomidine is a centrally acting *α*₂-adrenergic agonist that provides sedative, anxiolytic, and analgesic effects while typically preserving spontaneous respiration. Its mechanism of action involves the direct activation of α₂-adrenergic receptors, which can induce cerebral vasoconstriction and a consequent reduction in CBF—an effect also observed with other agents in this class, such as clonidine (39, 40). The impact of dexmedetomidine on CA appears to be nuanced. Evidence from studies suggests that while it does not impair static CA, it may compromise dynamic CA (41). However, the effect on dynamic CA remains a subject of controversy, as other investigations have reported divergent findings (42, 43). These inconsistencies may be attributable to confounding perioperative variables, such as patient positioning, or to differences in monitoring methodologies. Consequently, the net clinical value of dexmedetomidine’s effects on CA awaits clarification from future high-quality clinical studies and meta-analyses.

### 5.7 Opioids

Opioids modulate cerebral hemodynamics primarily through the activation of *μ*- and *κ*-opioid receptors, often resulting in region-specific increases in CBF (44). Despite these cerebrovascular effects, most opioids exhibit minimal direct impact on CA. Remifentanil, for instance, reduces ICP and CPP but leaves CA largely unaffected (45). Similarly, sufentanil shows no direct effect on autoregulatory function. Although morphine and fentanyl can increase ICP and lower MAP, thereby reducing CPP, they do not significantly alter CA indices, even in patients with severe brain injury (46).

A critical clinical consideration is the abrupt cessation of remifentanil. Sudden discontinuation can precipitate an acute withdrawal-like phenomenon characterized by sympathetic activation, leading to hypertension and tachycardia. In vulnerable patients with impaired intracranial compliance, these rapid hemodynamic swings may indirectly elevate ICP and challenge CA integrity, despite the drug’s lack of direct cerebrovascular effects. In summary, the influence of opioids on CA is predominantly indirect, mediated through their effects on systemic hemodynamics rather than via direct action on cerebrovascular regulation.

### 5.8 Barbiturates

Barbiturates represent a class of sedative-hypnotic agents whose use in clinical practice has declined. A key characteristic of these drugs is their ability to reduce CBF while simultaneously preserving CA. Evidence from animal models demonstrates that phenobarbital can restore CA that has been disrupted by seizures, underscoring its potential to stabilize cerebral hemodynamics under pathological conditions (47). These findings indicate that despite their suppressive effect on global CBF, barbiturates maintain the brain’s intrinsic capacity for pressure-flow regulation. This property historically underpinned their utility in neurocritical care, where the preservation of CA is a paramount objective.

### 5.9 Vasoactive agents

Vasoactive agents, which exert their primary effects on the cardiovascular system, can indirectly influence CA through hemodynamic alterations. The available evidence reveals agent-specific and context-dependent effects. For instance, whereas dopamine does not impair CA in animal models, it induces dose-dependent impairment in pediatric patients (48, 49). A recent meta-analysis indicates that norepinephrine significantly increases CBF in patients with pre-existing CA impairment, with the magnitude of increase correlating with the severity of autoregulatory dysfunction and the degree of MAP elevation (50). Direct evidence regarding phenylephrine’s effect on CA is currently lacking, though some studies note altered CA during phenylephrine-induced hypertension (51, 52). Ephedrine and dobutamine appear to have no significant impact on cerebral hemodynamics (53). Atropine has been shown to increase transfer function coherence in the very low-frequency range, a finding potentially confounded by postural changes during upright procedures (54). Collectively, these observations highlight that the cerebral effects of vasoactive agents are complex. A significant knowledge gap remains regarding their impact on CA in conscious, neurologically intact individuals.

### 5.10 Local anesthetics

Local anesthetics can influence CA when used in concentrations sufficient to produce sympathetic blockade. Direct stellate ganglion blockade, for example, typically results in a moderate but significant increase in ipsilateral CBF. The absence of this effect in some reports may be attributable to technically incomplete sympathetic blockade (18). Studies employing the ganglionic blocker trimetaphan have consistently demonstrated altered CA, supporting the interpretation that the sympathetic nervous system plays a critical role in its maintenance (55). Nevertheless, the precise mechanisms through which sympathetic blockade modulates CA remain incompletely elucidated.

## 6 Impact of surgical approach and duration on cerebral autoregulation

The nature and invasiveness of a surgical procedure are critical determinants of CA integrity. Minimally invasive surgeries, such as laparoscopic procedures, primarily challenge CA through indirect mechanisms. These include hypercapnia secondary to carbon dioxide pneumoperitoneum and alterations in venous return affecting ICP (56). In stark contrast, major surgeries—such as cardiac surgery requiring cardiopulmonary bypass, extensive tumor resections, and liver transplantation—pose a direct and profound threat to CA (57, 58). These interventions can trigger a systemic inflammatory response, precipitate significant fluid shifts, and increase the embolic burden. In the specific context of neurosurgery, direct mechanical manipulation of brain tissue presents an additional, potent insult. Consequently, these collective factors significantly elevate the incidence and severity of CA dysfunction. For instance, surgeries with high inherent risks of cerebral hypoperfusion or hyperperfusion—such as carotid endarterectomy, clipping of intracranial aneurysms, resection of large brain tumors (e.g., meningiomas or gliomas), and procedures performed in the sitting position (e.g., for posterior fossa lesions)—are among those that may derive the greatest benefit from continuous CA monitoring (59, 60).

## 7 The influence of surgical position on cerebral autoregulation

Patient positioning is a critical, yet frequently underappreciated, determinant of CA. Different surgical positions primarily affect CA by altering cerebral venous return and consequently influencing ICP. The Trendelenburg position, frequently employed in laparoscopic and robotic surgeries, elevates central venous pressure and ICP (56). This can challenge CA by reducing the effective CPP gradient, although the clinical significance of this effect remains a subject of ongoing discussion. The prone position, a mainstay in spinal and posterior fossa surgeries, requires meticulous execution. Improper positioning can compress abdominal vasculature, increase intra-abdominal pressure, and impede vena cava flow, thereby compromising cerebral venous return and elevating ICP. Direct evidence quantifying the impact of the prone position on CA is currently limited and warrants further investigation. The beach-chair position, utilized in shoulder and select posterior fossa procedures, carries a significant risk of orthostatic hypotension. This can drop MAP below the LLA, and studies have consistently documented impaired CA in this scenario (61). The interplay of positioning with other factors is particularly notable in spinal surgery. Here, the combined challenges of the prone position, potential for substantial blood loss, and the common anesthetic technique of controlled hypotension to minimize bleeding create a multifactorial threat to maintaining adequate CA. Furthermore, patients presenting with acute spinal cord injury may exhibit dysautonomia, which introduces secondary disturbances to systemic hemodynamics and cerebral autoregulatory capacity (7).

## 8 Clinical application and anesthetic management of perioperative monitoring of cerebral autoregulation function

Given the current technological limitations in continuously monitoring perfusion for most visceral organs, the strategy of using cerebral perfusion as a surrogate to guide systemic perfusion management has gained traction. This approach is physiologically grounded in the differential autoregulatory capacity across organ systems. The brain, spinal cord, heart, and kidneys possess robust autoregulation, whereas organs such as the stomach, intestines, liver, and pancreas exhibit weak or absent autoregulatory mechanisms (62). Consequently, these latter organs are at heightened risk of hypoperfusion during hemodynamic fluctuations. It is crucial to recognize that BP management targeting cerebral autoregulatory limits may still result in suboptimal perfusion of non-autoregulating organs. For instance, patients with significant coronary artery disease may require higher perfusion pressures than those sufficient for cerebral needs (63).

### 8.1 Evidence for CA-guided management

Accumulating evidence indicates that BP sustained below the LLA precipitates cerebral and systemic hypoperfusion, elevating the risk of end-organ injury and impairing functional recovery. Consequently, both impaired CA and BP below the LLA are associated with adverse clinical outcomes (2). Conversely, CA-guided BP management appears to simultaneously optimize cerebral perfusion and mitigate risks of systemic hypoperfusion. Maintaining perfusion pressure within the autoregulatory range, and ideally targeting the OptMAP, may thereby reduce hypoperfusion-related complications in vulnerable organ systems. The clinical benefits of this approach are particularly evident in cardiac surgery. Individualized BP management based on CA monitoring has been correlated with a reduced incidence of postoperative delirium and improved performance on memory tests (4). Furthermore, maintaining MAP above the LLA during cardiopulmonary bypass has been associated with a substantial reduction in delirium risk, though validation in broader surgical populations remains necessary (64).

The integration of CA monitoring into perioperative care requires a systematic, multi-faceted strategy:

- Target Definition: Establish individualized BP targets based on the patient-specific LLA.
- Anesthetic Selection: Carefully select anesthetic agents, prioritizing those known to preserve CA.
- Hemodynamic Management: Titrate vasoactive drugs in real-time to maintain MAP above the LLA threshold.
- Interdisciplinary Communication: Foster collaboration between anesthesiologists and surgeons to guide management during critical procedural phases.

Among available technologies, the NIRS-derived COx currently offers the most practical modality for routine perioperative use due to its non-invasive nature. TCD-based indices and the PRx remain valuable in specific, often more specialized, clinical contexts. The greatest utility for CA monitoring is anticipated in high-risk procedures—including cardiac, major vascular, neurosurgical, and trauma surgeries—where patients are most vulnerable to hemodynamic challenges. Ultimately, protocol-driven interventions, informed by real-time CA status and integrated with other monitoring parameters, represent the advancing frontier of precision hemodynamic management.

### 8.2 Limitations and future research directions

Despite its clinical relevance, the current evidence base on perioperative CA has notable and consequential limitations. Firstly, the overwhelming reliance on observational studies precludes definitive causal conclusions regarding the impact of CA impairment on patient outcomes. Secondly, the pronounced methodological heterogeneity in how CA is monitored and defined across studies severely impedes meaningful comparison and meta-analysis of findings. A major translational gap exists in the unresolved challenge of reliably determining individual-specific autoregulation thresholds at the bedside. Furthermore, our pharmacological understanding is incomplete and often contradictory, as exemplified by the marked heterogeneity in findings concerning dexmedetomidine’s effect on dynamic CA. Promising agents like xenon currently lack feasibility for widespread use. Critically, the confounding effects of ubiquitous perioperative factors (e.g., drug interactions, surgical stimulation) on CA indices have not been systematically evaluated, leaving a significant gap in our interpretative framework.

Future progress depends on addressing these gaps through targeted initiatives. Priority should be given to conducting multicenter randomized trials evaluating the impact of CA-guided management on patient-centered outcomes. Parallel efforts should focus on establishing standardized CA definitions and monitoring protocols. Technological innovation should aim to develop more automated systems, including closed-loop algorithms for BP control. Integrating combined CA with direct cortical electroencephalogram (dcEEG) monitoring and brain tissue partial pressure of oxygen (PbtO₂) monitoring into a multimodal framework may provide a more comprehensive assessment of brain function. It can also guide the real-time and personalized management of cerebral perfusion pressure and strengthen the closed-loop physiological control system in high-risk neurosurgical and intensive care settings. Further research is needed to elucidate the mechanisms of anesthetic-induced CA disruption and to assess the cost-effectiveness of CA monitoring in broader surgical populations, including elderly, pediatric, and critically ill patients.

## 9 Conclusion

Cerebral autoregulation is influenced by a complex interplay of anesthetic agents, surgical factors, and patient-specific conditions. Propofol, ketamine, and opioids tend to preserve CA, whereas volatile anesthetics may impair it in a dose-dependent manner. However, no single anesthetic technique guarantees CA integrity, as surgical challenges such as cardiopulmonary bypass or patient positioning can independently disrupt CA. Therefore, real-time CA monitoring may serve as a valuable adjunct to guide individualized blood pressure management (targeting LLA or OptMAP) rather than relying solely on anesthetic selection. A structured, multimodal approach that integrates CA monitoring with routine hemodynamic management is recommended. Future randomized trials are needed to validate its clinical utility and to establish standardized protocols.
